# Heliox preconditioning exerts pulmonary protection effects on neonatal acute respiratory distress syndrome by inhibiting oxidative stress and apoptosis

**DOI:** 10.3389/fphar.2025.1621190

**Published:** 2025-08-15

**Authors:** Juan Ma, Leilei Shen, Yuan Shi

**Affiliations:** ^1^ Department of Neonatology Children’s Hospital of Chongqing Medical University, National Clinical Research Center for Child Health and Disorders, Ministry of Education Key Laboratory of Child Development and Disorders, Chongqing Key Laboratory of Pediatric Metabolism and Inflammatory Diseases, Chongqing, China; ^2^ Department of Neonatology, SongShan General Hospital, Chongqing, China; ^3^ Department of Pediatrics, Third Military Medical University Southwest Hospital, Chongqing, China

**Keywords:** heliox preconditioning, neonatal acute respiratory distress syndrome, CaMK-II, anti-oxidative stress, anti-apoptosis

## Abstract

**Objective:**

This study aimed to investigate whether heliox preconditioning (HePC) alleviates neonatal acute respiratory distress syndrome (ARDS) by inhibiting oxidative stress and apoptosis, and to explore its potential mechanism.

**Methods:**

Blood samples and bronchoalveolar lavage fluid (BALF) were collected from rat pups were randomly divided into control group, sham group, ARDS group, ARDS + CaMKII^−^ group, ARDS + CaMKII^+^ group, and ARDS + HePC group. We also investigated the role of CaMKII by manipulating its expression *in vitro*. Inflammatory markers, oxidative stress, apoptosis and activation of signaling pathways were assessed using histological staining, ELISA, Western blotting, qRT-PCR, Ca^2+^, immunofluorescence staining, and flow cytometry.

**Results:**

*In vivo*, HePC significantly reduced the expression of CaMKII, inhibited the activation of CaMKII/RyR2, ameliorated the LPS-induced lung histopathological changes in rat pups, reduced lung wet/dry ratios, ROS and MDA levels, and pro-inflammatory cytokine levels, and significantly increased the expression of antioxidant proteins (Nrf2, HO-1 and SOD) and reduced LPS-induced apoptosis. *In vitro*, overexpression of CaMKII increases oxidative stress and activates RyR2, leading to cytoplasmic Ca^2+^ overload and increased apoptosis. HePC can reverse the above reactions by inhibiting the expression of CaMKII.

**Conclusion:**

HePC may attenuate oxidative stress through CaMKII and alleviate cytoplasmic Ca^2+^ overload by regulating CaMKII/RyR2, which inhibits apoptosis, exerting lung protection against ARDS.

## 1 Introduction

Acute respiratory distress syndrome (ARDS) is a common clinical critical disease and is one of the main causes of death and disability in neonates (Pediatric Acute Lung Injury Consensus Conference Group, 2015). As an acute diffuse inflammatory lung disease, the mortality rate can be as high as 20% or more (De Luca et al., 2022). Since the etiology and pathogenesis of ARDS have not been fully elucidated, there is currently a lack of targeted treatment options (De Luca et al., 2017). Compared with other age groups, neonatal ARDS is often superimposed on perinatal neonatal diseases, with more severe clinical symptoms, longer course, higher mortality, and often requires more advanced respiratory support and multi-organ comprehensive support (Chekole et al., 2023; Wang et al., 2015). Therefore, it is imperative to develop novel treatments for neonatal ARDS.

Heliox (a mixture of oxygen and helium) is an odorless, non-explosive, non-flammable gas. Its density is about 3 times lower than that of air and it is very safe (Szczapa et al., 2022). Heliox has been extensively employed in deep commercial and technical diving to mitigate nitrogen narcosis and reduce respiratory work at high ambient pressures (Lee et al., 2020). Clinically, heliox has demonstrated efficacy in managing acute upper airway obstruction (e.g., severe croup) and viral bronchiolitis in infants, where its low density reduces airway resistance and improves ventilation (Moraa et al., 2021; Liet et al., 2015). In recent years, a burgeoning body of research has demonstrated the organ-protective effects of heliox and heliox preconditioning (HePC) across a spectrum of animal experiments and clinical studies (Zhong et al., 2023; Smit et al., 2019). However, there are no relevant reports on the lung protective effect of HePC on neonatal ARDS.

Calcium/calmodulin-dependent protein kinase II (CaMKII) is a multifunctional serine/threonine kinase activated by calcium overload and oxidative stress. In pulmonary pathologies, CaMKII drives inflammation, apoptosis, and mitochondrial dysfunction by phosphorylating downstream targets like ryanodine receptors, exacerbating calcium dysregulation and cellular injury (Wang et al., 2021; Winters et al., 2016). Given its central role in stress signaling, we hypothesized CaMKII as a key mediator of neonatal ARDS pathogenesis and a potential target for HePC.

## 2 Materials and methods

### 2.1 Animals and grouping

Sprague-Dawley (SD) rat pups were purchased from Chongqing Medical University, China. A total of 48 7-day-old rat pups were used in this study. Neonatal rat pups were randomly assigned to six experimental groups (n = 8 per group) using computer-generated sequences: (1) Normal (untreated controls), (2) Sham (PBS-instilled), (3) ARDS (LPS-induced), (4) ARDS + CaMKII^−^ (CaMKII inhibited), (5) ARDS + CaMKII^+^ (CaMKII overexpression), and (6) ARDS + HePC (heliox preconditioning). Outcome assessors were blinded to treatment allocations during histological scoring, flow cytometry analysis, and physiological parameter measurements to minimize detection bias. The rats were housed at room temperature (22–25 °C) with a 12 h-light/dark cycle. Rats were randomly fed with standard chow and water and adapted to experimental conditions at least 3 days before the experiment. All experiments were carried out following the “Guidelines for the Use of Laboratory Animal Care” and approved by the Ethics Committee of Children’s Hospital of Chongqing Medical University (CHCMU-IACUC 20240508007), and animal sufering was minimized to the greatest extent possible.

### 2.2 Neonatal rat ARDS model

Seven-day-old Sprague-Dawley rat pups were anesthetized with 2% isoflurane (induction) followed by 1.5% maintenance (100% O_2_) using a neonatal facemask, with body temperature maintained at 37 °C via a heated pad to prevent hypothermia. LPS (5 mg/kg, Solarbio, L8880) or phosphate-buffered saline (PBS) (sham) was administered intratracheally under direct visualization: rats were placed supine with neck extended, the epiglottis gently depressed using blunt forceps to expose the glottis, and a blunted microneedle (0.5–1 cm insertion depth) was used for slow instillation (30 s injection). Rats were immediately suspended vertically for 30 s to ensure homogeneous pulmonary distribution. Hemodynamic stability was confirmed by non-invasive monitoring. Rats were monitored every 12 h for distress signs (dyspnea, lethargy, weight loss >15%); any animal meeting predefined humane endpoints (no pain reflex, rectal temperature <25 °C) received prompt euthanasia with pentobarbital overdose (150 mg/kg IP). Notably, all pups survived the 48-h observation period, consistent with prior reports of this LPS dosage in neonatal models (Ivanovski et al., 2023). Blood and BALF were collected at 48 h post-LPS for cytological and biochemical analyses. Lungs were harvested—right for histopathology (4% PFA fixation), left for wet-to-dry (W/D) ratio and molecular assays (snap-frozen in liquid N_2_)—following euthanasia confirmed by apnea and corneal pallor (>1 min).

### 2.3 HePC and interfering viruses administration

One day before intratracheal injection of LPS, rat pups were placed in a dedicated chamber at 37 °C and exposed to 70% helium-30% oxygen for three 5-min periods, interspersed with 5-min washout periods using air. Maintain normal pressure and temperature throughout the exposure period. This HePC scheme is one of the currently reported solutions with better protective effects (Zhong et al., 2023; Li et al., 2016a; Zhang et al., 2014). In addition, CaMKII inhibition/overexpression virus was intranasally instilled 4 days before intratracheal LPS injection in the ARDS + CaMKII^−^ group and ARDS + CaMKII^+^ group.

### 2.4 *In vitro* cell model

A549 cells were obtained from the China Cell Line Bank. The cells were cultured in medium (DMEM) supplemented with 10% fetal bovine serum (FBS) in a humidified incubator containing 5% CO_2_ at 37 °C. The cultured cells were randomly divided into seven groups: control group, LPS group, LPS + HePC group, LPS + HePC + Ad-null group, LPS + HePC + CaMKII^+^ group, LPS + HePC + Ad-null group, and LPS + HePC + CaMKII^−^ group. Except for the control group, cells were exposed to LPS (1 μg/mL) for 12 h, then the medium was replaced with DMEM and treated with a helium-oxygen mixture for 2 h, followed by the addition of CaMKII overexpression/inhibition viruses according to the group assignments. After a further 24 h of incubation, samples were collected for PCR analysis. The cells were then incubated for an additional 24 h, after which samples were collected for enzyme-linked immunosorbent assay (ELISA) and Western blot (WB) analysis.

### 2.5 H&E staining

The rat pups were sacrificed 48 h after LPS challenge. The right lung lobes were excised, fixed, embedded, and cut into 5 μm slices, then the sections were stained with hematoxylin and eosin. These sections were then meticulously examined under a light microscope.

### 2.6 Lung wet/dry (W/D) weight ratio

The left lung tissues were harvested, weighed (wet lung weight), dried in an oven at 60 °C for 72 h and weighed again (dry lung weight) to calculate the W/D ratios.

### 2.7 ELISA

ELISA was used to measure rat BALF, lung tissue, and cell supernatants. All procedures were performed according to the kit instructions (Supplementary Table S1). The optical density of each well was determined at a wavelength of 450 nm using a microplate reader. After obtaining the optical density value for each sample, the corresponding cytokine concentration was calculated based on the standard curve.

### 2.8 WB

Protein samples were extracted from rat lung tissues and A549 cells using radioimmunoprecipitation assay (RIPA) lysis buffer (Beyotime, China) containing protease inhibitor cocktail (cOmplete Mini, EDTA-free; Roche, Switzerland). PBMCs were isolated by Ficoll density gradient centrifugation (800 × g, 20 min), while lung tissues were pulverized in liquid nitrogen and homogenized in RIPA buffer (100 mg tissue/mL). After ice-cold lysis (30 min) and centrifugation (12,000 × g, 15 min, 4 °C), protein concentrations were determined by bicinchoninic acid (BCA) assay (Thermo Fisher Scientific, United States) using bovine serum albumin (BSA; Sigma-Aldrich, United States) as a standard. Proteins (20 µg/lane) were separated on 6%–15% sodium dodecyl sulfate-polyacrylamide gel electrophoresis (SDS-PAGE) gels and transferred to polyvinylidene fluoride (PVDF) membranes (0.45 µm; Millipore, United States) at 100 V for 30–240 min (target-optimized). Membranes were blocked with 5% non-fat dried milk (NFDM; Yili Group, China) in Tris-buffered saline with Tween-20 (TBST; 10 mM Tris, 150 mM NaCl, 0.1% Tween-20, pH 7.5) and incubated overnight at 4 °C with primary antibodies (see Supplementary Table S1), followed by horseradish peroxidase (HRP)-conjugated secondary antibodies (1:1,000; Zhongshan Golden Bridge, China). Protein bands were visualized using enhanced chemiluminescence (ECL; Tanon 5,200, China) and quantified by ImageJ software (National Institutes of Health, United States), normalized to β-actin.

### 2.9 qRT-PCR

Total RNA was isolated from rat lung tissue and cells using TRIzol reagent. The extracted RNAs were reverse transcribed into complementary DNA and real-time PCR was performed to semiquantitatively analyse CaMKII, ryanodine receptor 2 (RyR2), BCL2-Associated X protein (Bax) and B-cell lymphoma-2 (Bcl2) expression in each sample.

The qPCR reaction consisted of a denaturation step of 95 °C for 6 min, followed by 40 cycles of denaturation at 95 °C for 30 s and annealing at 57 °C for 30 s. Primer information is in Supplementary Table S2.

### 2.10 Measurement of oxygen species (ROS) production

For the measurement of reactive ROS, cells were collected from rat BALF and A549 cells. Cells that were not subjected to any treatment were resuspended in 0.01 M PBS and designated as the negative control. Cells were resuspended in diluted DCFH-DA, and a hydrogen peroxide donor was added to induce ROS production, serving as the positive control. Sample tubes were prepared by resuspending cell pellets in diluted DCFH-DA. The cells were incubated at 37 °C for 1 h. Following incubation, the single-cell suspensions (stained with the probe) were collected, centrifuged, and the cell pellets were used for fluorescence detection. The results were expressed as fluorescence intensity values.

### 2.11 Determination of malondialdehyde (MDA) contents

The BALF and cells were homogenized and levels of MDA were measured using test kits according to the manufacturer’s instructions.

### 2.12 Immunofluorescence labeling

Lung tissue sections were fixed with 4% paraformaldehyde (P0099, Beyotime), dehydrated through xylene (3 × 10 min) and ethanol gradients (100%, 90%, 85%; 8 min each), then subjected to antigen retrieval in citrate buffer (microwave heating: medium power 10 min, standing 10 min, medium-low power 8 min). After blocking with serum for 60 min at room temperature, sections were incubated overnight at 4 °C with rabbit anti-CaMKII (1:100, 13730-1-AP, Proteintech), followed by FITC-conjugated goat anti-rabbit IgG (1:100, ZF0311, Zhongshan Golden Bridge) for 60 min at room temperature in the dark. Nuclei were counterstained with DAPI (C1005, Beyotime) and slides were mounted with anti-fade mounting medium. Images were acquired using a fluorescence microscope (MSHOT, Guangzhou) with consistent exposure settings across samples.

Intracellular Ca^2+^ levels were measured using Fluo-4 a.m. calcium assay kit (S1061, Beyotime, China). Briefly, A549 cells seeded on coverslips were washed with PBS (pH 7.4) and incubated with Fluo-4 a.m. staining solution (250 μL/well for 24-well plate, containing 0.2 μM Fluo-4 a.m. and 0.2 μM solubility enhancer in assay buffer) at 37 °C for 30 min in the dark. After washing with PBS, cells were imaged immediately using a fluorescence microscope (MF53, Mshot, China) with FITC filter settings (excitation/emission: 490/525 nm) and a ×63 oil immersion objective. Image acquisition parameters (exposure time/gain) were kept consistent across all experimental groups to enable quantitative comparison.

Apoptosis was detected using the *In Situ* Cell Death Detection Kit (C1090, Beyotime, China). Tissue sections were deparaffinized, rehydrated, and subjected to antigen retrieval. After blocking, sections were incubated with transferase dUTP-mediated nick-end labeling (TUNEL) reaction mixture (50 μL/section) overnight at 37 °C, followed by DAPI counterstaining (C1005, Beyotime). Images were captured using a fluorescence microscope (MF53, Mshot, China). Negative controls (omitting TdT enzyme) confirmed assay specificity.

### 2.13 Flow cytometry

Apoptosis was quantified using the Annexin V-FITC/PI Apoptosis Detection Kit (C1052, Beyotime, China). Briefly, A549 cells (1 × 10^6^ cells/sample) were digested with 0.25% EDTA-free trypsin (Solarbio, T1300), washed twice with ice-cold PBS (pH 7.4), and resuspended in 195 μL binding buffer. Cells were stained with 5 μL Annexin V-FITC and 10 μL PI (20 μg/mL final concentration) for 15 min at 25 °C in the dark, followed by immediate analysis using a BD Accuri C6 flow cytometer (BD Biosciences) with 488 nm (FITC) and 561 nm (PI) excitation. Data from ≥10,000 events were analyzed using FlowJo V10 (BD Biosciences), with unstained and single-stained controls for compensation.

### 2.14 Statistical analysis

Statistical analyses were performed using SPSS version 23.0. Descriptive statistics were calculated, with quantitative variables presented as mean ± standard deviation and qualitative variables as proportions. Differences between continuous variables were assessed using one-way ANOVA, with *post hoc* comparisons using the LSD test for equal variances and Dunnett’s T3 test for unequal variances. Graphs, including histograms, were generated using GraphPad Prism version 8.0. Statistical significance was set at *P* values less than 0.05, with P values less than 0.01 or 0.001 indicating high significance, indicated as *P < 0.05, **P < 0.01 and ***P < 0.001, respectively.

## 3 Results

### 3.1 HePC protects against LPS-induced lung injury and inflammation

**FIGURE 1:**
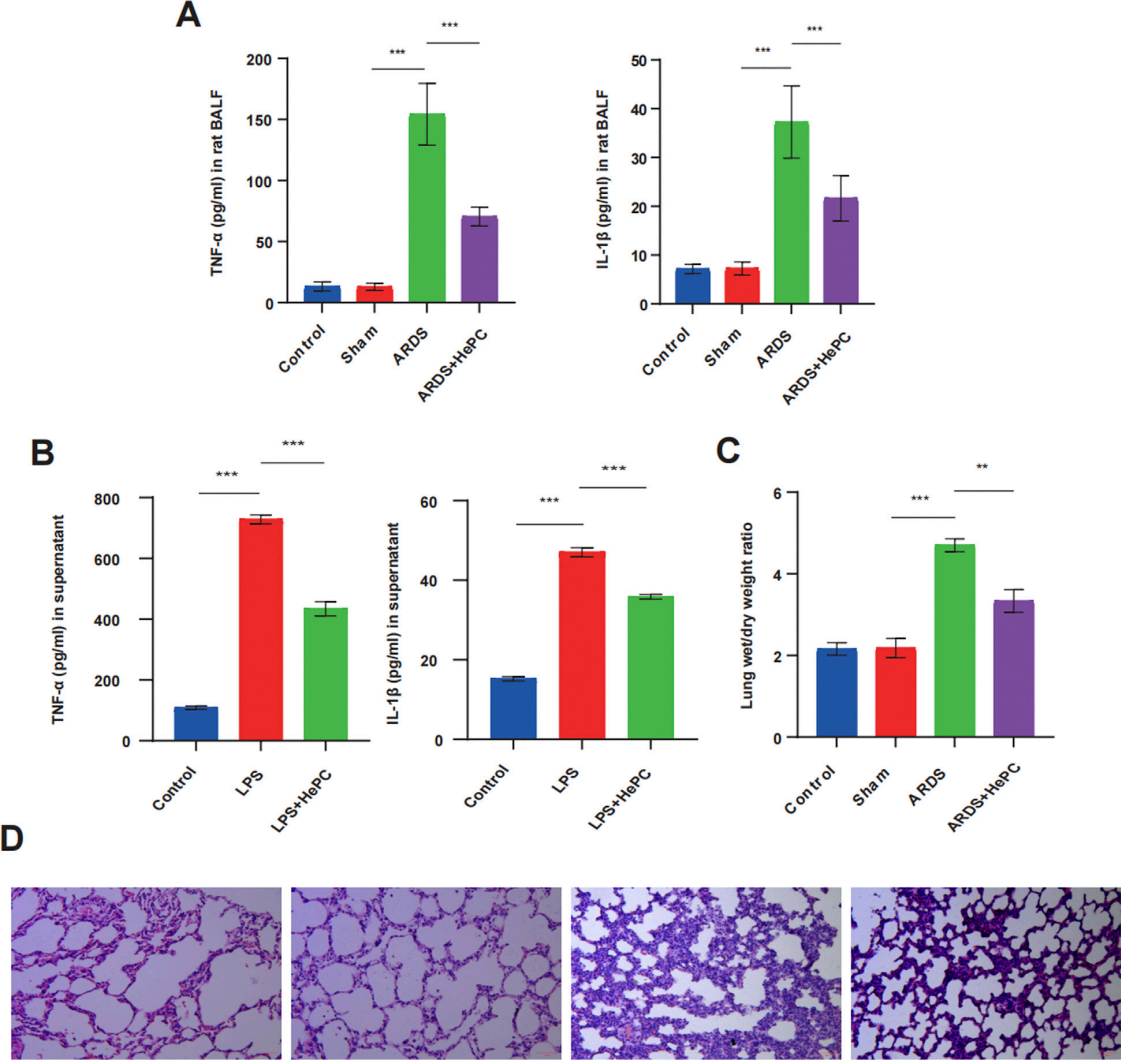
HePC protects against LPS-induced lung injury and inflammation. **(A)** TNF-α and IL-1β levels in rat BALF. **(B)** TNF-α and IL-1β levels in the cell supernatant. **(C)** Lung wet/dry weight ratios were evaluated at 48 h post LPS treatment. **(D)** H&E staining of lung sections (×100). **P < 0.01, ***P < 0.001.

To investigate the potential effects of HePC on LPS-induced ARDS in rat and cellular models, rats were exposed to a helium-oxygen mixed gas environment 1 day prior to LPS infusion, and cells were incubated with LPS for 12 h followed by treatment with a helium-oxygen mixture for 2 h. Then, lung tissue, BALF, and cell supernatant were collected to evaluate the severity of lung injury, pro-inflammatory cytokines, and lung W/D weight ratio. Rat developed ARDS with obvious lung tissue alterations at 48 h after LPS insult. Compared to the control group and sham group, the ARDS group presented destructive alveolar structure, thickened alveolar septum, increased inflammatory cells infiltration, diffuse alveolar and interstitial edema (Figure 1D). The expression levels of pro-inflammatory cytokines TNF-α and IL-1β in BALF and cell supernatants increased (Figures 1A,B), and the lung W/D ratio increased (Figure 1C). Notably, these lung histopathological alterations were significantly attenuated by HePC, as shown with rough alveolar structure, mild inflammatory cells infiltration and pulmonary edema (Figure 1D), and the expressions of TNF-α and IL-1β were also attenuated by HePC (Figures 1A,B).

### 3.2 HePC counteracts LPS-induced oxidative stress via inhibiting CaMKII

**FIGURE 2:**
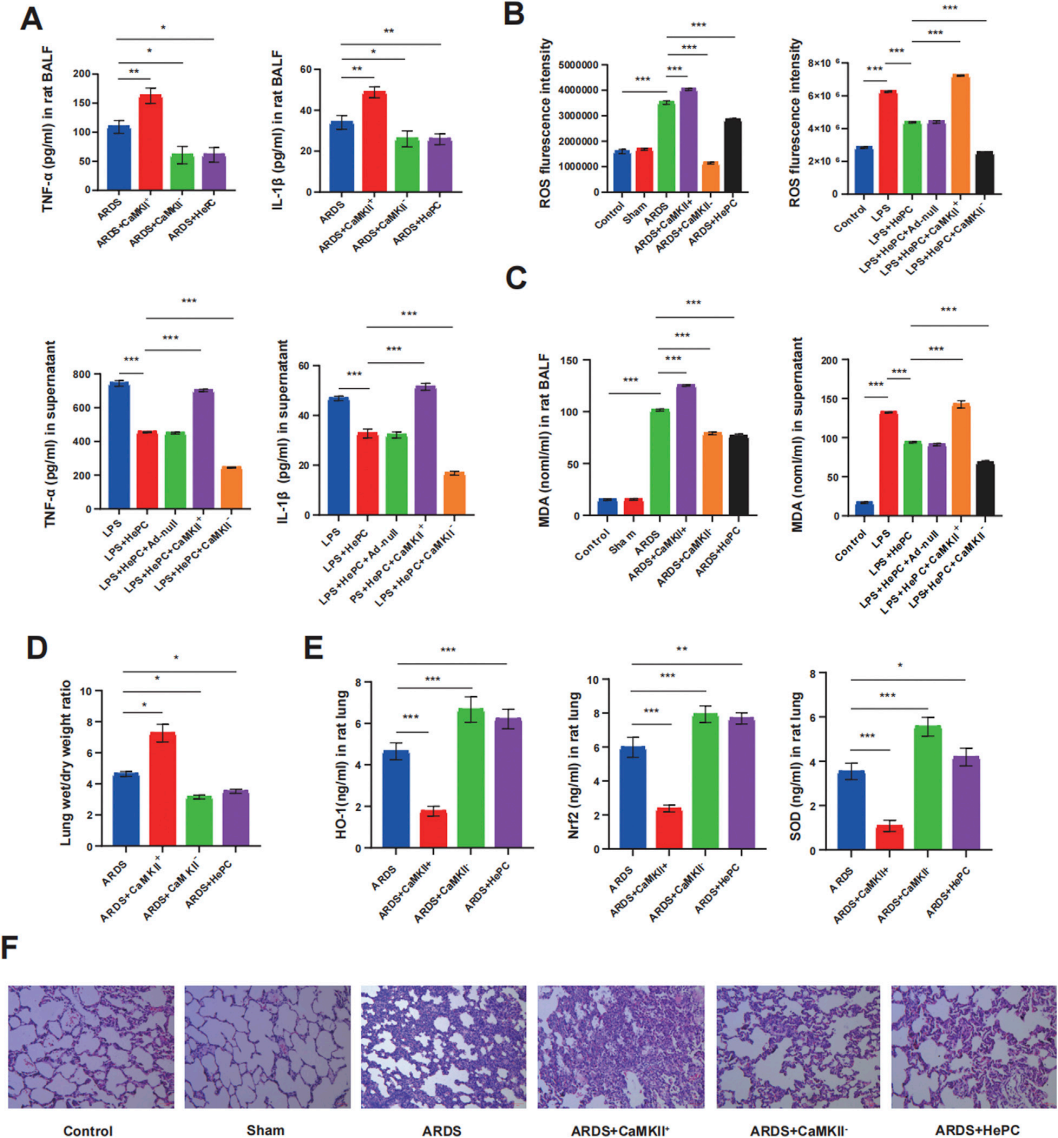
HePC ameliorates pulmonary inflammation via promoting antioxidant defenses. **(A)** TNF-α and IL-1β levels in rat BALF. **(B)** Quantitative ROS fluorescence intensity of each group in rats and cell models. **(C)** MDA levels in both rat BALF and cell supernatants. **(D)** Lung wet/dry weight ratios were evaluated at 48 h post LPS treatment. **(E)** HO-1, Nrf2 and SOD levels in rat lung. **(F)** H&E staining of lung sections (×100). *P < 0.05, **P < 0.01, and ***P < 0.001.

We evaluated the impact of HePC on LPS-induced oxidative stress and the expression of antioxidant proteins, as well as the potential regulatory mechanisms involved. HePC significantly reduced the production of ROS and MDA induced by LPS in rat lung tissue and A549 cells (Figures 2B,C). In contrast, HePC markedly increased the expression of antioxidants (heme oxygenase-1, HO-1, and superoxide dismutase, SOD) and enhanced cellular redox balance and the expression of the antioxidant transcriptional upstream regulator Nrf2 (Figure 2E).

**FIGURE 3:**
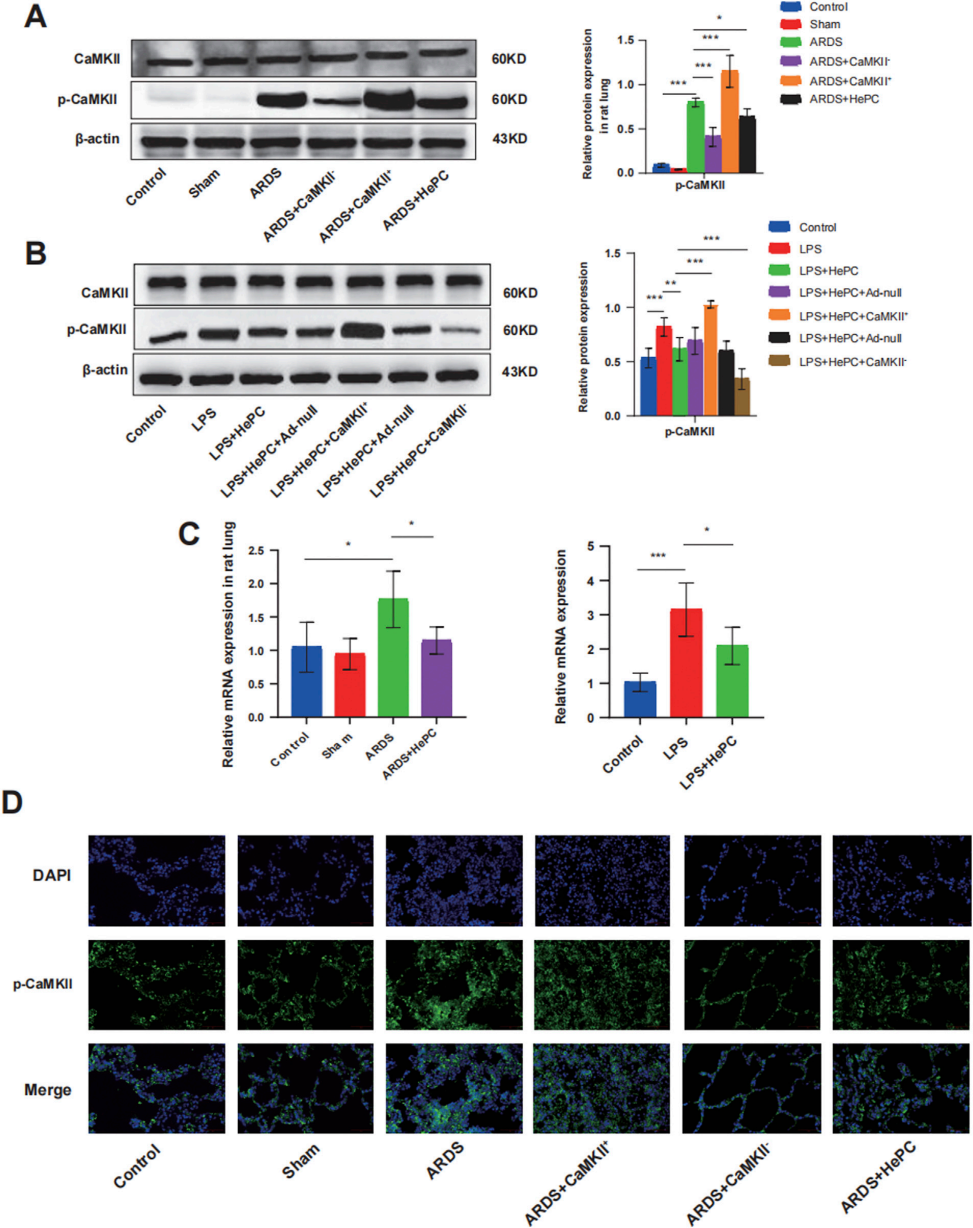
HePC Regulates the Expression of CaMKII. **(A)** Representative protein blot images of CaMKII and p-CaMKII in rat lung. Data are presented as mean ± SD (n = 5 per group). **(B)** Representative protein blot images of CaMKII and p-CaMKII in cells. Data are presented as mean ± SD (n = 5 per group). **(C)** Expression of CaMKII mRNA in rat lung and cell were detected by qRT-PCR. Values are means ± SD (n = 5 per group). **(D)** Immunofluorescence for p-CaMKII (green) in rat lung sections. Nuclei stained with DAPI (blue) (scale bar = 50 μm). *P < 0.05, **P < 0.01, and ***P < 0.001.

To further elucidate the underlying mechanisms by which HePC alleviates oxidative stress, we examined CaMKII and found that its expression was significantly enhanced in rats and cellular models, with increased phosphorylation levels, indicating an activated state (Figures 3A–D). Additionally, the regulation of CaMKII has a potential impact on the release of pro-inflammatory cytokines. Analysis of BALF and cell supernatants revealed that overexpression of CaMKII leads to elevated levels of pro-inflammatory factors, while suppression of CaMKII expression can reduce pro-inflammatory factor levels (Figure 2A). Furthermore, modulating CaMKII affects the balance between oxidants (ROS and MDA) and antioxidants (HO-1, SOD) and their upstream regulator Nrf2, influencing the extent of pulmonary edema and lung tissue inflammatory damage. Enhanced expression of CaMKII results in increased expression of ROS and MDA (Figures 2B,C), reduced expression of HO-1, SOD, and Nrf2 (Figure 2E), increased lung W/D ratio (Figure 2D), and further exacerbation of lung tissue inflammatory damage (Figure 2F), while suppression of CaMKII expression can reverse these changes. Notably, the regulatory role of CaMKII in oxidative stress is influenced by HePC, which significantly reduces the LPS-induced increase in p-CaMKII levels (Figures 3A,B,D), decreases the release of pro-inflammatory factors (Figure 2A), reduces the expression of oxidants (ROS and MDA) (Figures 2B,C), increases the expression of tested antioxidants (HO-1, SOD) (Figure 2E), and alleviates lung tissue W/D ratio (Figure 2D) and inflammatory damage (Figure 2F).

### 3.3 The effects of HePC on LPS-induced apoptosis in ARDS

**FIGURE 4:**
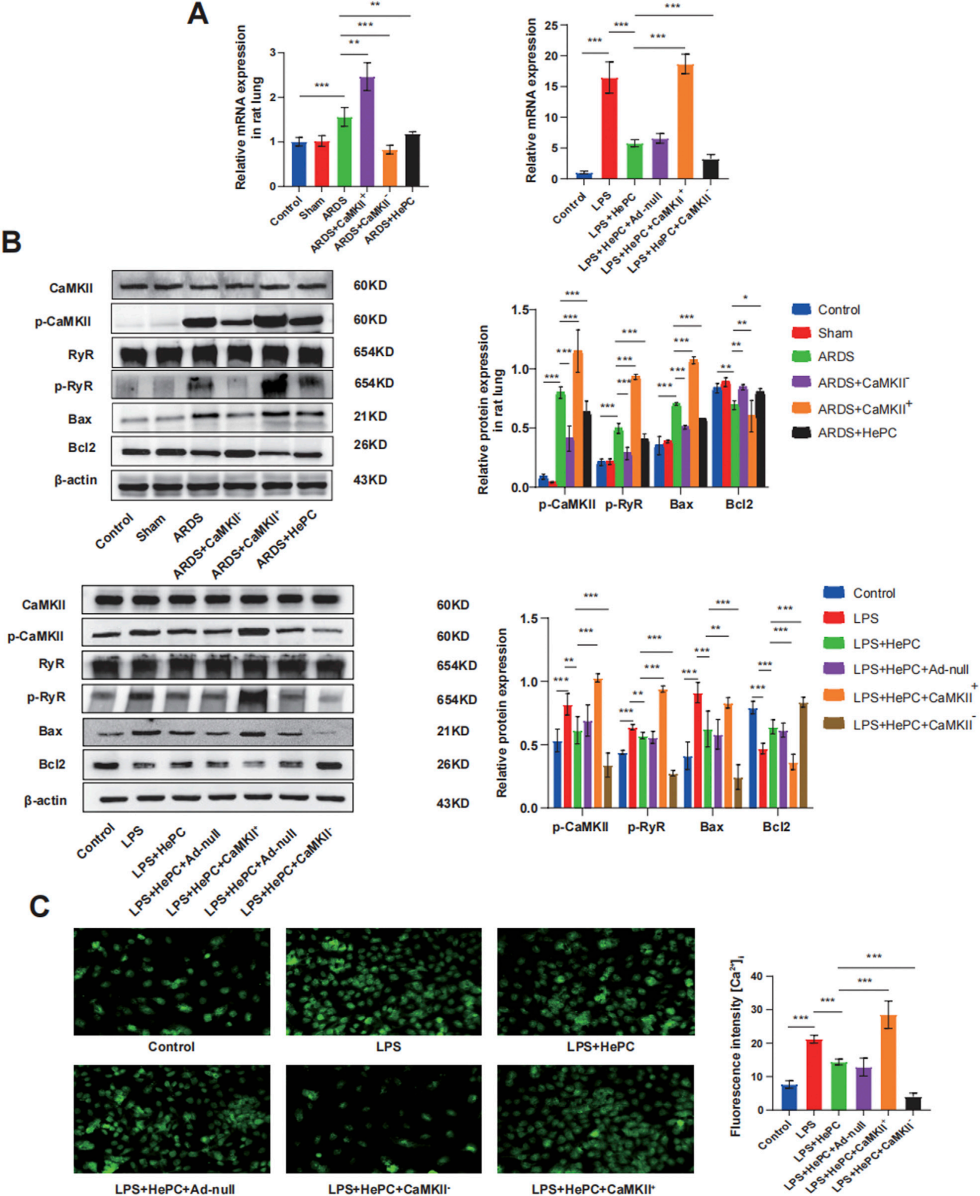
HePC Regulates Apoptosis through the CaMKII/RyR2 Signaling Pathway. **(A)** Expression of RyR mRNA in rat lung and cell were detected by qRT-PCR. Values are means ± SD (n = 5 per group). **(B)** Representative protein blot images in rat lung and cells. Data are presented as mean ± SD (n = 5 per group). **(C)** Calcium ionophore response in A549 lung carcinoma cells loaded with Fluo-4. Representative figure from nine experiments. *P < 0.05, **P < 0.01, and ***P < 0.001.

**FIGURE 5:**
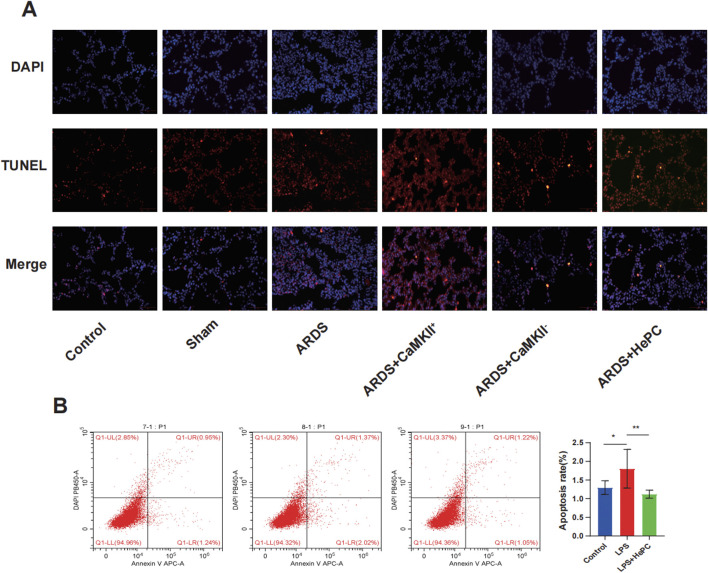
The effects of HePC on LPS-Induced Apoptosis. **(A)** Representative images of TUNEL assay staining to detect apoptotic cells in the lung sections of rat at 48 h. Scale bar = 50 μm. **(B)** The apoptosis of A549 cells was detected by flow cytometry *in vitro*. *P < 0.05, **P < 0.01.

To investigate whether HePC has a protective effect against LPS-induced apoptosis, we examined the levels of Bax and Bcl2 proteins and preliminarily explored the underlying mechanisms. We found that compared to the control and sham groups, the ARDS group exhibited significantly increased levels of p-RyR2 and Bax proteins, decreased levels of Bcl2 protein (Figure 4B), and markedly elevated mRNA levels of RyR2 (Figure 4A). HePC effectively reduced the LPS-induced increase in protein expression (p-RyR2, Bax) and enhanced the expression of the anti-apoptotic protein Bcl2 (Figure 4B). Consistently, TUNEL assays showed an increase in apoptosis following LPS-induced injury, and HePC significantly reduced the number of TUNEL-positive cells (Figure 5A). *In vitro* cellular experiments demonstrated that flow cytometry analysis indicated HePC reduced the rate of apoptosis (Figure 5B).

Mechanistic exploration revealed that enhanced expression of CaMKII led to further increases in p-RyR2 and Bax protein levels, decreased Bcl2 protein levels (Figure 4B), significantly elevated RyR2 mRNA levels (Figure 4A), increased intracellular calcium ion levels (Figure 4C), and increased apoptosis at the animal level (Figure 5A). Inhibition of CaMKII significantly suppressed the expression of p-RyR2/RyR2 and Bax (Figure 4B), reduced cell apoptosis (Figure 5A), and upregulated the expression of Bcl2 (Figure 4B). HePC, by inhibiting the expression of CaMKII, led to reduced expression of RyR2 and Bax, enhanced expression of Bcl2 (Figure 4B), decreased intracellular calcium ion levels (Figure 4C), and ultimately significantly inhibited cell apoptosis stimulated by LPS (Figures 5A,B).

## 4 Discussion

This study demonstrates that HePC exerts pulmonary protection in neonatal ARDS by downregulating CaMKII-mediated oxidative stress and apoptosis. Our multimodal approach revealed: (1) rat models showed HePC reduced LPS-induced lung injury and oxidative stress; (2) *in vitro* studies demonstrated HePC reversed Ca^2+^ overload and apoptosis via CaMKII inhibition. Notably, the rapid protective effects of short-duration HePC observed in our experiments may involve membrane stabilization and ion channel modulation, as suggested by prior studies showing helium’s ability to attenuate Ca^2+^ leakage through RyR2 phosphorylation suppression within minutes (Zhong et al., 2023). This non-genomic mechanism aligns with the clinical feasibility of brief preconditioning protocols, though longer exposure durations might further enhance efficacy by potentially activating additional anti-inflammatory pathways—a hypothesis requiring future validation. These consistent findings across systems validate HePC’s therapeutic potential through conserved mechanisms, following established respiratory research paradigms (Boutanquoi et al., 2020; Hu et al., 2022; Wang et al., 2025). To our knowledge, this represents the first mechanistic evidence of HePC’s protection in neonatal ARDS.

Increased oxidative stress, characterized by an imbalance between oxidants and antioxidants in favor of the oxidants, is a major contributing factor to the pathogenesis of ARDS (Cannavò et al., 2021). In this study, the expression of ROS and MDA was significantly increased in rats and A549 cells, indicating a marked elevation in mitochondrial oxidative stress. HePC reversed the increase in the oxidative stress marker MDA and significantly enhanced the expression of endogenous antioxidants (Nrf2, HO-1, and SOD), leading to a reduction in pro-inflammatory cytokines and alleviation of pulmonary inflammatory damage. This aligns with clinical observations that heliox ventilation reduces systemic inflammation in neonates with obstructive airway disorders, as demonstrated by Szczapa et al. (Szczapa et al., 2022) in their review of neonatal applications. Since ROS can upregulate the expression of pro-inflammatory cytokines, exacerbating tissue damage and pulmonary edema, maintaining an appropriate oxidant-antioxidant balance is crucial for the treatment of ARDS (Dhlamini et al., 2022). Our study suggests that HePC may counteract LPS-induced oxidative stress by enhancing endogenous antioxidant defenses. Furthermore, multiple studies (Nawab et al., 2005; Wise et al., 2018) have indicated that heliox breathing may have combined therapeutic benefits of attenuating lung inflammation, yet the underlying molecular mechanisms remain elusive. Recent clinical trials by Ma et al. (2021) further support that heliox reduces inflammatory markers in neonatal with meconium aspiration syndrome, corroborating our findings. Therefore, it is imperative to elucidate the molecular mechanisms of HePC in ARDS pulmonary protection, thereby paving the way for novel therapeutic strategies.

CaMKII is a multifunctional serine/threonine protein kinase regulated by calcium/calmodulin complexes (Zhang et al., 2023). According to previous literature reports (Zhang et al., 2016; Adameova et al., 2022), CaMKII triggers the opening of the mitochondrial permeability transition pore and myocardial necroptosis through phosphorylation, oxidation, or both, CaMKII inhibition has been shown to alleviate oxidative stress and apoptosis by restoring IRE1α/XBP1s signaling (Kong et al., 2022), yet its role in modulating the redox reactions in ARDS has not been previously documented. Our study found that HePC may regulate oxidative stress by modulating CaMKII. The expression of oxidative and antioxidative markers, as well as the levels of inflammatory cytokines, undergo corresponding changes with the modulation of CaMKII. HePC significantly inhibits the phosphorylation of CaMKII, leading to a reduction in LPS-induced production of TNF-α and IL-1β in rats. Similar results were obtained in the cultured human epithelial cell line A549, further validating the anti-inflammatory effect mediated by HePC through the suppression of CaMKII activity, consistent with the protective mechanisms of CaMKII inhibition in polycystic kidney diseases models involving oxidative stress and apoptosis (Bracken et al., 2016).

Prior research has posited that the apoptosis of pulmonary endothelial cells could initiate a sequence of pathophysiological events culminating in the development of ARDS (Galani et al., 2010). Studies have reported that activated CaMKII can phosphorylate the RyR and calcium pumps, exacerbating intracellular calcium overload and leading to cardiomyocyte apoptosis (Popescu et al., 2016; Li et al., 2016b). Our research findings indicate that in ARDS, CaMKII similarly affects apoptosis by regulating the phosphorylation of RyR2, while HePC alleviates intracellular calcium overload by inhibiting CaMKII and demonstrates an inhibitory effect on apoptosis at both animal and cellular levels. Growing evidence suggests that reactive ROS are crucial for inducing cellular apoptosis (Simon et al., 2000; Wang et al., 2023; Jiang et al., 2022); ROS can reduce the expression of Bcl2 and increase the expression of Bax, thereby affecting mitochondrial membrane permeability and triggering cell apoptosis (Guo et al., 2023). Furthermore, during the process of apoptosis, the production of ROS increases, leading to heightened oxidative stress (Zhang et al., 2022). Apoptosis can lead to increased oxidative stress, and this heightened oxidative stress, in turn, can promote the progression of apoptosis, creating an interactive network between the two (Wang QQ et al., 2022; Wang Y et al., 2022). HePC, by regulating CaMKII, can simultaneously suppress oxidative stress and apoptosis.

## 5 Limitations

This study has several limitations inherent to its preclinical design. Most notably, ethical constraints preclude clinical validation of helium-oxygen mixtures in neonatal ARDS patients, necessitating caution when extrapolating our rodent and cellular findings to human pathophysiology. Furthermore, while we identified CaMKII/RyR2 as a key regulatory axis, the molecular landscape remains incompletely mapped—critical calcium regulators such as the inositol 1,4,5-trisphosphate receptor were not investigated, and potential synergism between HePC and direct CaMKII modulation (e.g., combined HePC + CaMKII inhibition) warrants dedicated exploration. Future studies should prioritize human-relevant models to confirm translational applicability and dissect additional mechanisms, including combinatorial therapeutic strategies and parallel calcium signaling pathways.

## 6 Conclusion

This study demonstrates that HePC protects against LPS-induced ARDS by inhibiting oxidative stress and apoptosis through CaMKII. These findings provide preliminary experimental evidence for the potential application of HePC in the treatment of ARDS.

## Data Availability

The datasets presented in this study can be found in online repositories. The names of the repository/repositories and accession number(s) can be found in the article/Supplementary Material.
