# Supplementary material for: Heliox preconditioning exerts pulmonary protection effects on neonatal acute respiratory distress syndrome by inhibiting oxidative stress and apoptosis
Source: Front Pharmacol. 2025 Aug 15;16:1621190. doi: 10.3389/fphar.2025.1621190 (PMC12394224; doi:10.3389/fphar.2025.1621190)
Supplement: Supplementary file 1 [file Supplementaryfile1.docx]

**Table 1 Key Resource Table**

**I. Antibodies**

| **Target** | **Host** | **Catalog No. (Dilution)** | **Vendor** |
| --- | --- | --- | --- |
| **Primary Antibodies** |  |  |  |
| CaMKII | Rabbit | 13730-1-AP (1:3000) | Proteintech, Wuhan, China |
| p-CaMKII | Rabbit | AF7378 (1:1000) | Affinity, Nanjing, China |
| RyR2 | Rabbit | Ab219798 (1:1000) | Abcam, Cambridge, UK |
| p-RyR2 | Rabbit | AF7454 (1:1000) | Affinity, Nanjing, China |
| Bax | Rabbit | AF0120 (1:2000) | Affinity, Nanjing, China |
| Bcl2 | Rabbit | AF6139 (1:1000) | Affinity, Nanjing, China |
| β-actin | Mouse | SC-47778 (1:1000) | Santa Cruz, CA, USA |
| **Secondary Antibodies** |  |  |  |
| Goat anti-rabbit IgG-HRP | Goat | ZB-2301 (1:1000) | Zhongshan Golden Bridge, Beijing, China |
| Goat anti-mouse IgG-HRP | Goat | ZB-2305 (1:1000) | Zhongshan Golden Bridge, Beijing, China |
| Goat anti-rabbit FITC | Goat | ZF0311 (1:200) | Zhongshan Golden Bridge, Beijing, China |

**II. Kits & Assays**

| **Application** | **Catalog No.** | **Vendor** |
| --- | --- | --- |
| **ELISA Kits** |  |  |
| Human IL-1β | JL13662 | JiangLai, Shanghai, China |
| Rat IL-1β | JL20884 | JiangLai, Shanghai, China |
| Human TNF-α | JL10208 | JiangLai, Shanghai, China |
| Rat TNF-α | JL13202 | JiangLai, Shanghai, China |
| Rat HO-1 | JL10795 | JiangLai, Shanghai, China |
| Rat Nrf2 | JL13922 | JiangLai, Shanghai, China |
| Rat SOD | JL22893 | JiangLai, Shanghai, China |
| **Oxidative Stress Assays** |  |  |
| MDA Content | A003-1 | Nanjing Jiancheng Bioengineering, Nanjing, China |
| ROS Detection | E004-1-1 | Nanjing Jiancheng Bioengineering, Nanjing, China |
| **Molecular Biology** |  |  |
| ReverTra Ace qPCR RT Kit | FSQ-101 | TOYOBO, Osaka, Japan |
| TRNzol Reagent | DP424 | TIANGEN, Beijing, China |
| **Cell Staining** |  |  |
| Fluo-4 AM Calcium Assay | S1061 | Beyotime, Shanghai, China |
| TUNEL Apoptosis Detection | C1090 | Beyotime, Shanghai, China |
| HE Staining Kit | C0105 | Beyotime, Shanghai, China |
| DAPI Staining Kit | C1005 | Beyotime, Shanghai, China |
| **Flow Cytometric** |  |  |
| Annexin V-FITC/PI Apoptosis Detection Kit | C1052 | Beyotime, Shanghai, China |
| **Protein Quantification** |  |  |
| BCA Protein Assay Kit | P0012 | Beyotime, Shanghai, China |
| **ECL Reagents** |  |  |
| ECL Substrate | P0018 | Beyotime, Shanghai, China |
| **SDS-PAGE** |  |  |
| Prestained Protein Marker | PR1910 | Beyotime, Shanghai, China |
| **Reagent** |  |  |
| RIPA Lysis Buffer | P0013B | Beyotime, Shanghai, China |
| Bovine Serum Albumin | A1938 | Sigma-Aldrich, St. Louis, USA |
| Lipopolysaccharide | L8880 | Solarbio, Beijing, China |

**III. Instruments**

| **Instrument** | **Model** | **Vendor** |
| --- | --- | --- |
| PCR System | 7500 | Thermo Fisher Scientific, MA, USA |
| Spectrophotometer | SmartSpec 3000 | Bio-Rad, Hercules, USA |
| Optical Microscope | Imager A2 | ZEISS, Oberkochen, Germany |
| Fluorescence Microscope | MF53 | Mshot, Guangzhou, China |
| Centrifuge | TG16-WS | Xiangyi, Changsha, China |
| Flow Cytometer | Accuri C6 | BD Biosciences, San Jose, USA |
| Microplate Reader | FLvostar Omega | BMG Labtech, Offenburg, Germany |
| Chemiluminescence Imaging System | Tanon 5200 | Tanon Science & Technology, Shanghai, China |
| Vertical Electrophoresis System | EPS300 | Tanon, Shanghai, China |

**Table 2 qRT-PCR primer sequences used in this study**

| Gene | Sequence (5’‐ 3’) |
| --- | --- |
| CaMKII | F：5'- AACCCTCACATCCACCTG -3' |
|  | R：5'- ATCTGCCATTTTCCATCC -3' |
| RyR2 | F：5'- CGCATCCTAGCCATCCTCC -3' |
|  | R：5'- GCCACTTCCTTCTCTCGC -3' |
| β-actin | F：5'-ACCCCGTGCTGCTGACCGAG-3' |
|  | R：5'-TCCCGGCCAGCCAGGTCCA-3' |
